# mTOR has a developmental stage-specific role in mitochondrial fitness independent of conventional mTORC1 and mTORC2 and the kinase activity

**DOI:** 10.1371/journal.pone.0183266

**Published:** 2017-08-16

**Authors:** Khalid W. Kalim, Shuangmin Zhang, Xiaoyi Chen, Yuan Li, Jun-Qi Yang, Yi Zheng, Fukun Guo

**Affiliations:** 1 Division of Experimental Hematology and Cancer Biology, Children’s Hospital Research Foundation, Cincinnati, Ohio, United States of America; 2 Key Laboratory for Parasitic Disease Control and Prevention, Ministry of Health, Jiangsu Institute of Parasitic Diseases, Wuxi, Jiangsu, China; University of South Alabama, UNITED STATES

## Abstract

The mammalian target of rapamycin (mTOR), present in mTOR complex 1 (mTORC1) and mTORC2, is a serine/threonine kinase that integrates nutrients, growth factors, and cellular energy status to control protein synthesis, cell growth, survival and metabolism. However, it remains elusive whether mTOR plays a developmental stage-specific role in tissue development and whether mTOR can function independent of its complexes and kinase activity. In this study, by inducible genetic manipulation approach, we investigated the role of mTOR and its dependence on mTOR complexes and kinase activity in mitochondrial fitness of early, progenitor stage (lineage-negative; Lin^-^) versus later, lineage-committed stage (lineage-positive; Lin^+^) of hematopoietic cells. We found that oxidative phosphorylation (OXPHOS), ATP production and mitochondrial DNA synthesis were decreased in mTOR^-/-^ Lin^-^ cells but increased in mTOR^-/-^ Lin^+^ cells, suggesting that mTOR plays a developmental stage-specific role in OXPHOS, ATP production and mitochondrial DNA synthesis. In contrast to mTOR deletion, simultaneous deletion of Raptor, a key component of mTORC1, and Rictor, a key component of mTORC2, led to increased mitochondrial DNA in Lin^-^ cells and decreased mitochondrial DNA and ATP production in Lin^+^ cells, suggesting that mTOR regulates mitochondrial DNA synthesis in Lin^-^ and Lin^+^ cells and ATP production in Lin^+^ cells independent of mTORC1 and mTORC2. Similar to mTOR deletion, deletion of Raptor alone attenuated glycolysis and increased mitochondrial mass and mitochondrial membrane potential in Lin^-^ cells and increased mitochondrial mass and OXPHOS in Lin^+^ cells, whereas deletion of Rictor alone had no effect on these mitochondrial parameters in Lin^-^ and Lin^+^ cells, suggesting that mTOR regulates glycolysis and mitochondrial membrane potential in Lin^-^ cells, OXPHOS in Lin^+^ cells, and mitochondrial mass in both Lin^-^ and Lin^+^ cells dependent on mTORC1, but not mTORC2. Either Raptor deficiency or Rictor deficiency recapitulated mTOR deletion in decreasing OXPHOS in Lin^-^ cells and glycolysis in Lin^+^ cells, suggesting that mTOR regulates OXPHOS in Lin^-^ cells and glycolysis in Lin^+^ cells dependent on both mTORC1 and mTORC2. Finally, mice harboring a mTOR kinase dead D2338A knock-in mutant showed decreased glycolysis in Lin^+^ cells, as seen in mTOR^-/-^ Lin^+^ cells, but no change in glycolysis in Lin^-^ cells, in contrast to the decreased glycolysis in mTOR^-/-^ Lin^-^ cells, suggesting that mTOR regulates glycolysis in Lin^+^ cells dependent on its kinase activity, whereas mTOR regulates glycolysis in Lin^-^ cells independent of its kinase activity.

## Introduction

The mammalian target of Rapamycin (mTOR) is a serine/threonine kinase that is present in two molecular complexes: mTOR complex 1 (mTORC1) and mTORC2. mTORC1 is comprised of Raptor, mLST8, PRAS40 and Deptor, and mTORC2 is comprised of Rictor, mLST8, mSIN1 and Protor [1]. In response to extracellular stimuli such as nutrients and growth factors and intracellular stimuli such as elevated energy state, mTORC1 is activated by phosphatidylinositol-3-OH (PI 3) kinase, PDK1 and Akt [1, 2]. Activated mTORC1 regulates ribosome biogenesis, protein synthesis, cell growth, and autophagy through S6K1 and 4E-BP. mTORC2, on the other hand, promotes cell survival through Akt and/or PKC-θ [2, 3]

Mitochondria are the main site for generating ATP, the major energy source for many cellular activities including cell proliferation and survival. Mitochondrial ATP production is driven by mitochondrial metabolism such as oxidative phosphorylation (OXPHOS) and glycolysis [4]. Proper mitochondrial function relies on the homeostatic maintenance of mitochondrial mass, mitochondrial membrane potential and mitochondrial DNA contents. It is well known that mitochondrial metabolism is regulated by mTOR [4, 5]. mTORC1 signaling regulates glucose, glutamine, lipid, amino acid and nucleic acid metabolism in a variety of cells including fibroblasts, T lymphocytes, epithelial cells and hepatocytes [6–9]. Although less studied, mTORC2 is also shown to regulate glycolysis, lipogenesis and OXPHOS in hepatocytes [4, 10, 11]

It is generally thought that mTOR acts through mTOR complexes. In support, mTOR regulates oxidative muscle integrity via mTORC1 and endothelial cell proliferation through both mTORC1 and mTORC2 [12, 13]. However, it remains unknown whether mTOR has a complex-independent role in regulating cell behaviors. mTOR kinase activity is presumably required for mTOR function. In this aspect, mTOR kinase activity is responsible for phosphorylation of S6K, 4E-BP and AKT, and chemical inhibitors of mTOR kinase activity suppress normal and cancer cell growth and/or survival [14–16]. However, genetic evidence of the importance of mTOR kinase activity is lacking and it is elusive whether mTOR has kinase activity-independent function. Moreover, it remains unclear whether mTOR plays a developmental stage-specific role in tissue development. In this study, by single deletion of mTOR, Raptor or Rictor, simultaneous deletion of Raptor and Rictor, and knock-in of a mTOR kinase dead (KD) mutant, we have examined mTOR complex and kinase activity dependence of mTOR in the regulation of mitochondrial fitness (e.g. ATP production, OXPHOS, glycolysis, mitochondrial mass, mitochondrial membrane potential and mitochondrial DNA synthesis) of hematopoietic cells. We have found that mTOR may regulate mitochondrial fitness dependent or independent of mTOR complexes and kinase activity. By comparing the effects of mTOR deletion on mitochondrial fitness of early, progenitor stage (lineage-negative; Lin^-^) with that of later, lineage-committed stage (lineage-positive; Lin^+^) of hematopoietic cells, we show that mTOR has a developmental stage-specific role in regulating mitochondrial fitness.

## Materials and methods

### Mice

Animal study was in compliance with the Cincinnati Children’s Hospital Medical Center Animal Care and Use Committee protocols and with National Institutes of Health guide for the care and use of Laboratory animals (NIH Publications No. 8023, revised 1978). Animal study was performed under specific ethics approval obtained from the Cincinnati Children’s Hospital Medical Center Animal Care. Conditional gene-targeted *mTOR*^*loxp/loxp*^, *Raptor*^*loxp/loxp*^ and *Rictor*^*loxp/loxp*^ mice were generated as described previously [12, 17, 18]. The flox allele contains loxp sites flanking exon 1–5 of the *mTOR* gene, exon 6 of the *Raptor* gene, or exon 3 of the *Rictor* gene. To delete *mTOR*, *Raptor*, or *Rictor in vivo* in hematopoietic cells, *mTOR*^*loxp/loxp*^;*Mx-Cre*^+^, *Raptor*^*loxp/loxp*^; *Mx-Cre*^+^ or *Rictor*^*loxp/loxp*^;*Mx-Cre*^+^ mice were generated by breeding *mTOR*^*loxp/loxp*^, *Raptor*^*loxp/loxp*^ or *Rictor*^*loxp/loxp*^ mice with the *Mx-Cre*^+^ transgenic mice carrying a bacteriophage Cre recombinase driven by an interferon-α-inducible *Mx1* promoter. The expression of Cre was induced by 5 i.p. injections of polyinosine-polycytidine (poly I:C) (Amersham Pharmacia Biotech, Piscataway, NJ) into the *Mx-Cre*^+^ mice at 2-day intervals with each injection of 10 μg/g of body weight. Control, *Mx-Cre*^-^ mice also received poly I:C injections. The mice were utilized 7–10 days after last poly I:C injection. mTOR KD mutant D2338A knock-in mice were generated by CRISPR/Cas9 technology. Because *mTOR*^*KD/KD*^ mice were embryonically lethal and *mTOR*^*+/KD*^ mice didn’t show abnormalities [19], we generated *mTOR*^*loxp/KD*^ mice and crossed with *Mx1-Cre*^+^ mice. When the resultant *mTOR*^*loxp/KD*^*;Mx1-Cre*^+^ mice are treated with poly I:C, mTOR is ablated from the floxed allele. Thus, mTOR kinase activity is not only lost on the D2338A mutant knock-in allele but also on the floxed allele. All of the mice were housed under specific pathogen-free conditions in the animal facility at Cincinnati Children's Hospital Research Foundation. Mice were anesthetized when necessary, using ketamine (80–100 mg/kg im), aceptromazine (4–6 mg/kg im) and atropine (0.1mg/kg im). Anesthesia was maintained using ketamine (30 mg/kg im) as needed. During the course of experiments, mice were isolated in microisolator cages and cared for in the Laboratory Animal Resource Center by a trained technician and two veterinarians. Animals were checked daily by qualified personnel in the lab. The method of euthanasia used was CO_2_ euthanasia. The animal study was in compliance with the Cincinnati Children’s Hospital Medical Center Animal Care and Use Committee protocols and with the National Institutes of Health guide for the care and use of Laboratory animals (NIH Publications No. 8023, revised 1978)

### Immunoblot

Bone marrow cells were lysed and protein content was normalized by Bradford assay. Lysates were separated by 10% SDS-polyacrylamide gel electrophoresis. The expression of Raptor and Rictor and phosphorylation of S6K, 4E-BP and Akt (S473) were probed by Western blot using corresponding antibodies (Cell Signaling Technology).

### Analysis of OXPHOS and glycolysis

Single cell suspensions were prepared from bone marrow. Cells were then fractionated into Lin^-^ and Lin^+^ cells using a Lineage Cell Depletion Kit (Miltenyi Biotec), according to the manufacturer’s protocols. For measurement of OXPHOS, 10^6^ Lin^-^ and Lin^+^ cells were resuspended in XF assay medium (pH 7.4) containing GlutaMax (2 mM), sodium pyruvate (1 mM) and glucose (25 mM), plated onto Seahorse Bioscience XF24 cell culture plates coated with Cell Tak (BD Bioscience), and incubated without CO_2_ at 37°C. Oxygen consumption rate (OCR) was measured using the Seahorse XF24 Analyzer (Agilent Technologies, Santa Clara, CA) in the presence or absence of Oligomycin (0.6 μM), FCCP (1 μM), and Antimycin (1 μM) and Rotenone (1 μM). For measurement of glycolysis, 10^6^ Lin^-^ and Lin^+^ cells were resuspended in assay medium (pH 7.4) (Sigma-Aldrich) containing L-Glutamine (2 mM), plated onto XF24 cell culture plates, and incubated as for OCR. Extracellular acidification rate (ECAR) was measured using the Seahorse XF24 Analyzer in the presence or absence of Glucose (10 mM), Oligomycin and 2-DG [20, 21].

### Transcript expression analysis

RNA was isolated from Lin^-^ and Lin^+^ cells using RNeasy Micro Kit (QIAGEN) and converted to cDNA using a High Capacity cDNA Reverse Transcription Kit (Applied Biosystems Inc). Quantitative Real-time PCR was performed with SYBR Green dye or the Taqman assay on a 7900HT Real-Time machine (Applied Biosystems Inc, Foster City, CA). Data were analyzed using SDS 2.3 software (Applied Biosystems Inc, Foster City, CA) and normalized to GAPDH.

The primer sequences were as following: Nrf1: TATGGCGGAAGTAATGAAAGACG (forward), CAACGTAAGCTCTGCCTTGTT (reverse); Atp5I: GAGAAGGCACCGTCGATGG (forward), ACACTCTGAATAGCTGTAGGGAT (reverse); Cox5a: GCCGCTGTCTGTTCCATTC (forward), GCATCAATGTCTGGCTTGTTGAA (reverse); Ndufa2: TTGCGTGAGATTCGCGTTCA (forward), ATTCGCGGATCAGAATGG GC (reverse); HK2 F: TGATCGCCTGCTTATTCACGG (forward), AACCGCCTAGAAATCTCCAGA (reverse); slc2a: CAGTTCGGCTATAACACTGGTG (forward), GCCCCCGACAGAGAAGATG (reverse); PDK1: GGACTTCGGGTCAGTGAATGC (forward), TCCTGAGAAGATTGTCGGGGA (reverse); Hif1α: AGCTTCTGTTATGAGGCTCACC (forward), TGACTTGATGTTCATCGTCCTC (reverse).

### Analysis of mitochondrion mass and mitochondrial membrane potential

Bone marrow cells were incubated for 20 min at room temperature with antibodies against lineage markers Gr1, Mac1, Ter119, B220, CD3, CD4, and CD8 (BD Biosciences). For the measurement of Mitochondrion mass and mitochondrial membrane potential, the cells were incubated with 100 nM Mitotracker Green and 50 nM DilC-5 (Invitrogen), respectively, according to the manufacturer’s protocols. The Immunolabeled cells were analyzed by flow cytometry on a FACSCanto system using FACSDiVa software (BD Biosciences, Franklin Lakes, NJ) [21].

### Analysis of mitochondrial DNA

Purified Lin^-^ and Lin^+^ cells were lysed and homogenized and mitochondrial DNA was then purified with a Mitochondrial DNA isolation Kit (Biovision), according to the manufacturer’s manual. The purified mitochondrial DNA was quantified by quantitative real-time PCR with SYBR Green dye. Mitochondrial DNA content was represented by mitochondrial cyclo-oxygenase (Cox) 2 normalized to the nuclear intron of β-globin. The primer sequences were: Cox2: GCCGACTAAATCAAGCAACA (forward), CAATGGGCATAAAGCTATGG (reverse); β-globin: GAAGCGATTCTAGGGAGCAG (forward), GGAGCAGC GATTCTGAGTAGA (reverse) [22].

### Analysis of ATP

Purified Lin^-^ and Lin^+^ cells were lysed with a cytosol extraction buffer supplied in the Mitochondrial DNA isolation Kit, homogenized and then ATP was determined by an ATP Determination Kit (Molecular Probes), according to the manufacturer’s protocol.

### Statistical analysis

The data are expressed as the mean ± standard deviation (SD). The data were analyzed by Student’s unpaired t-test for single comparisons and by one-way ANOVA followed by Bonferroni test for multiple comparisons. Significance was accepted at p<0.05.

## Results

### A role of mTOR, mTORC1 and mTORC2 in OXPHOS, glycolysis and ATP production

**Fig 1:**
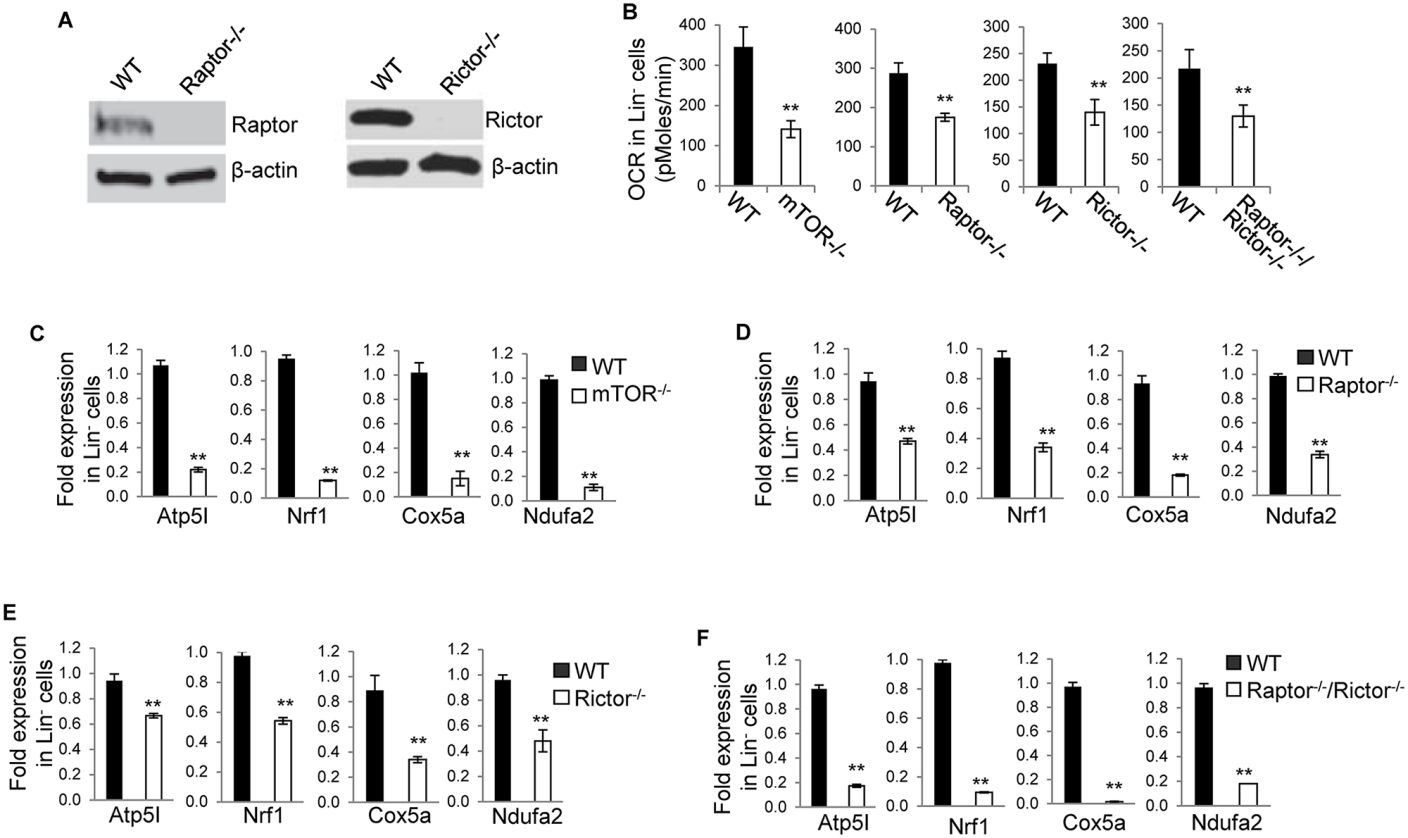
mTOR promotes OXPHOS in Lin^-^ cells depending on both mTORC1 and mTORC2. (A) Raptor and Rictor protein expression. Bone marrow cells were harvested from the indicated mice and detected for Raptor and Rictor expression by Western blot. β-actin was blotted as a loading control. (B-F) Bone marrow cells were harvested from the indicated mice. Lin^-^ cells were fractionated from the bone marrow cells and assayed for oxygen consumption rate (OCR) (B) and Atp5I, Nrf1, Cox5a, and Ndufa32 by Quantitative Real-time PCR (C-F). For (C-F), the mRNA expression levels were normalized to one WT mouse. Results are representative of three independent experiments. Error bars represent mean ± SD of 5–8 mice. **P < .01.

**Fig 2:**
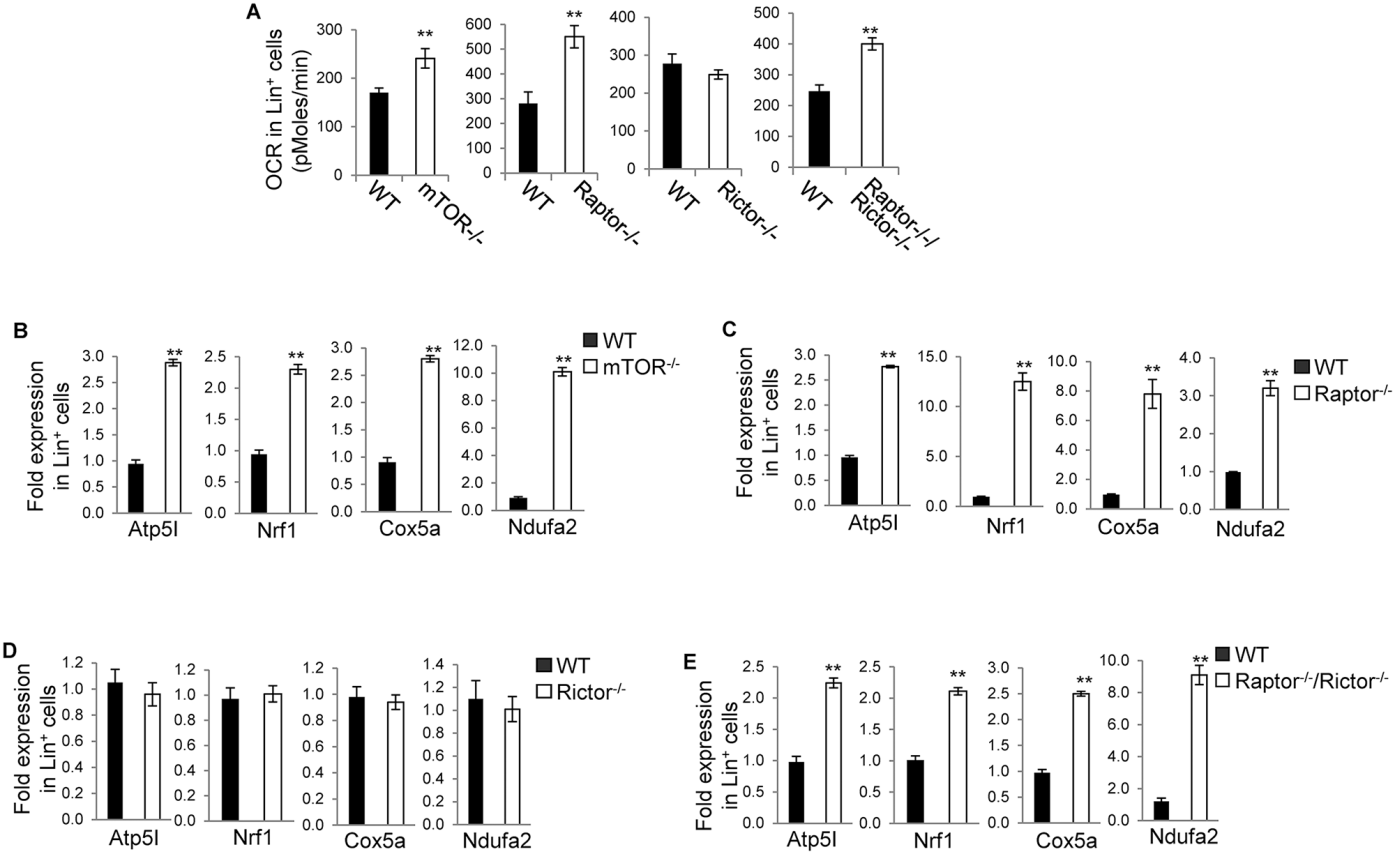
mTOR inhibits OXPHOS in Lin^+^ cells dependent on mTORC1 but not mTORC2. Bone marrow cells were harvested from the indicated mice. Lin^+^ cells were fractionated from the bone marrow cells and assayed for oxygen consumption rate (OCR) (A) and Atp5I, Nrf1, Cox5a, and Ndufa32 by Quantitative Real-time PCR (B-E). For (B-E), the mRNA expression levels were normalized to one WT mouse. Results are representative of three independent experiments. Error bars represent mean ± SD of 5–8 mice.. **P < .01.

To define the role of mTOR, mTORC1 and mTORC2 in mitochondrial fitness, we deleted *mTOR* in adult *mTOR*^*loxp/loxp*^*;Mx1-Cre*^+^ mice (*mTOR*^*-/-*^), *Raptor* in *Raptor*^*loxp/loxp*^*;Mx1-Cre*^+^ mice (*Raptor*^*-/-*^), *Rictor in Rictor*^*loxp/loxp*^*;Mx1-Cre*^+^ mice (*Rictor*^*-/-*^), or *Raptor* and *Rictor* in *Raptor*^*loxp/loxp*^*;Rictor*^*loxp/loxp*^*;Mx1-Cre*^+^ mice (*Raptor*^*-/-*^; *Rictor*^*-/-*^), by injection of polyinosine-polycytidine (poly I:C). Immunoblotting confirmed deletion of mTOR, Raptor, or Rictor in bone marrow cells of the respective mice (Fig 1A) [23]. OXPHOS in *mTOR*^*-/-*^, *Raptor*^*-/-*^, *Rictor*^*-/-*^, and *Raptor*^*-/-*^; *Rictor*^*-/-*^ Lin^-^ and Lin^+^ bone marrow cells and control, wild type (WT) cells were then examined by analysis of OCR. As shown in Fig 1B, deletion of mTOR, Raptor, or Rictor, or simultaneous deletion of Raptor and Rictor all led to a reduced OXPHOS in Lin^-^ cells. Consistently, Atp5a, Nrf1, Cox5, and Ndufa2, all of which are involved in regulating OXPHOS, were decreased in *mTOR*^*-/-*^, *Raptor*^*-/-*^, *Rictor*^*-/-*^, and *Raptor*^*-/-*^; *Rictor*^*-/-*^ Lin^-^ cells (Fig 1C–1F). While deletion of mTOR or Raptor, or simultaneous deletion of Raptor and Rictor caused an increased OXPHOS in Lin^+^ cells, deletion of Rictor had no effect on OXPHOS in Lin^+^ cells (Fig 2A). In line with this, Atp5a, Nrf1, Cox5, and Ndufa2 were increased in *mTOR*^*-/-*^, *Raptor*^*-/-*^, and *Raptor*^*-/-*^; *Rictor*^*-/-*^ Lin^+^ cells but not changed in *Rictor*^*-/-*^ Lin^+^ cells (Fig 2B–2E). These results suggest that mTOR promotes OXPHOS in Lin^-^ cells depending on both mTORC1 and mTORC2 but inhibits OXPHOS in Lin^+^ cells dependent on mTORC1 only. Thus, mTOR plays a developmental stage-specific role in regulating OXPHOS.

**Fig 3:**
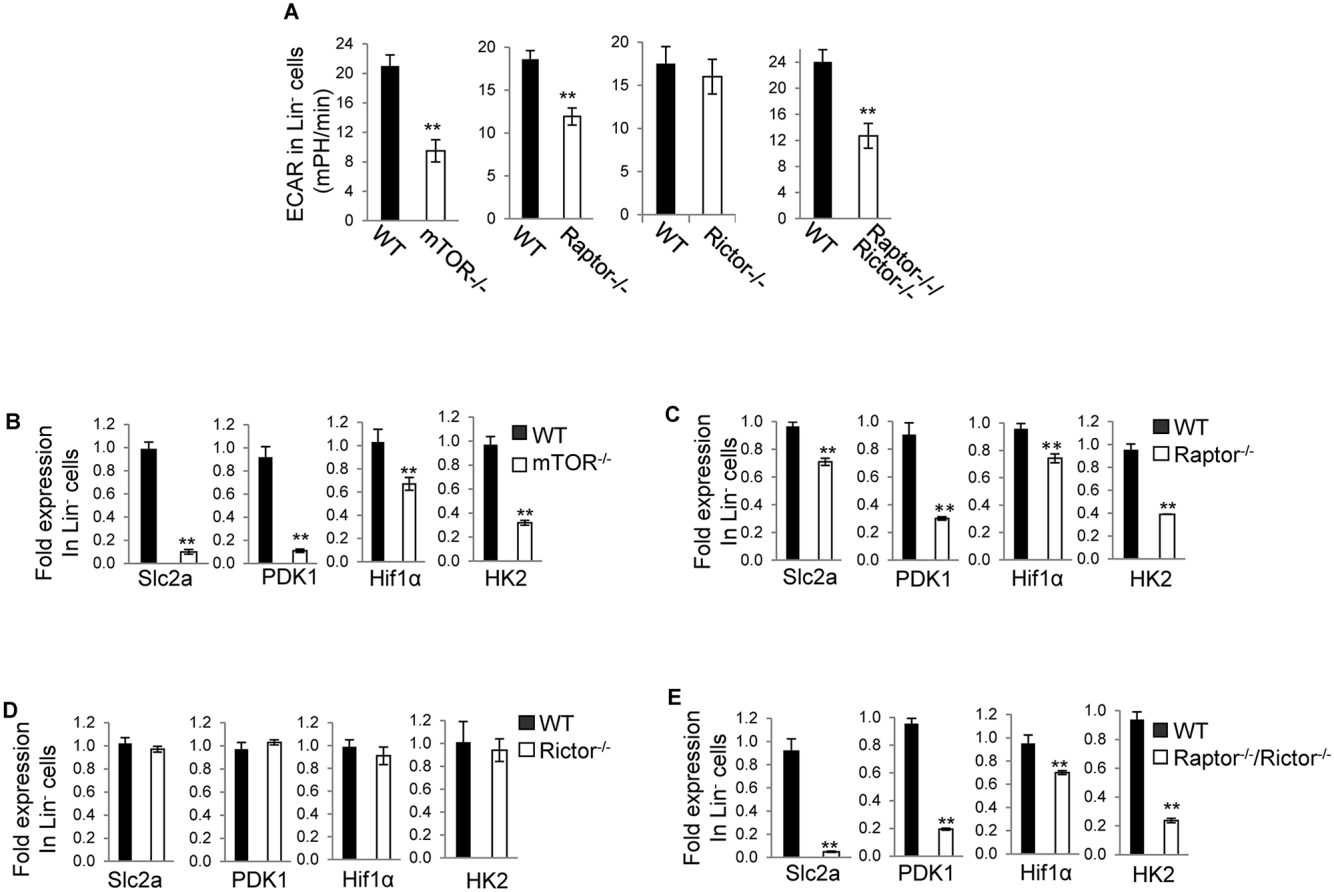
mTOR promotes glycolysis in Lin^-^ cells depending on mTORC1 but not mTORC2. Bone marrow cells were harvested from the indicated mice. Lin^-^ cells were fractionated from the bone marrow cells and assayed for extracellular acidification rate (ECAR) (A) and Slc2a, PDK1, Hif1α, and HK2 by Quantitative Real-time PCR (B-E). For (B-E), the mRNA expression levels were normalized to one WT mouse. Results are representative of three independent experiments. Error bars represent mean ± SD of 5–8 mice. **P < .01.

**Fig 4:**
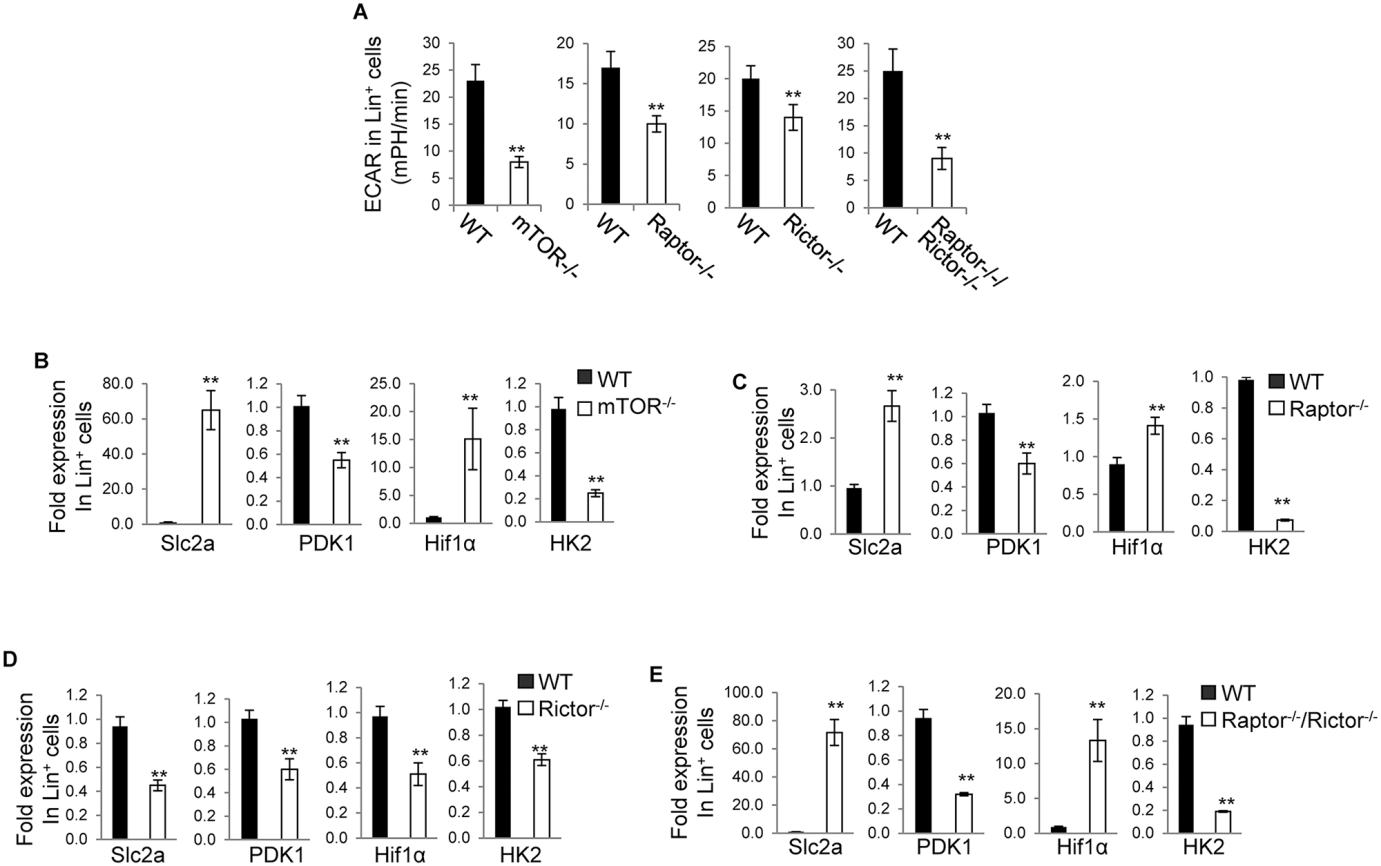
mTOR promotes glycolysis in Lin^+^ cells depending on both mTORC1 and mTORC2. Bone marrow cells were harvested from the indicated mice. Lin^+^ cells were fractionated from the bone marrow cells and assayed for extracellular acidification rate (ECAR) (A) and Slc2a, PDK1, Hif1α, and HK2 by Quantitative Real-time PCR (B-E). For (B-E), the mRNA expression levels were normalized to one WT mouse. Results are representative of three independent experiments. Error bars represent mean ± SD of 5–8 mice. **P < .01.

Next we detected glycolysis in *mTOR*^*-/-*^, *Raptor*^*-/-*^, *Rictor*^*-/-*^, *Raptor*^*-/-*^; *Rictor*^*-/-*^ and WT Lin^-^ and Lin^+^ cells by analysis of ECAR. Depletion of mTOR or Raptor or simultaneous deletion of Raptor and Rictor attenuated glycolysis in Lin^-^ cells. In contrast, deletion of Rictor had no effect on glycolysis in Lin^-^ cells (Fig 3A). In agreement with this, Slc2a, PDK1, Hif1α, and HK2, all of which are involved in regulating glycolysis, were decreased in *mTOR*^*-/-*^, *Raptor*^*-/-*^, and *Raptor*^*-/-*^; *Rictor*^*-/-*^ Lin^-^ cells but not changed in *Rictor*^*-/-*^ Lin^-^ cells (Fig 3B–3E). Deletion of mTOR, Raptor, or Rictor, or simultaneous deletion of Raptor and Rictor all resulted in a reduction of glycolysis in Lin^+^ cells (Fig 4A). In accordance with this, PDK1 and HK2 were decreased in *mTOR*^*-/-*^, *Raptor*^*-/-*^, and *Raptor*^*-/-*^; *Rictor*^*-/-*^ Lin^+^ cells and Slc2a, PDK1, Hif1α, and HK2 were decreased in *Rictor*^*-/-*^ Lin^+^ cells (Fig 4B–4E). Surprisingly, Slc2a and Hif1α were increased in *mTOR*^*-/-*^, *Raptor*^*-/-*^, and *Raptor*^*-/-*^; *Rictor*^*-/-*^ Lin^+^ cells (Fig 4B, 4C and 4E), which could reflect a compensatory effect of the decreased glycolysis. Thus, mTOR promotes glycolysis in both Lin^-^ and Lin^+^ cells. Nonetheless, it appears that mTOR regulates glycolysis in Lin^-^ cells through mTORC1 but not mTORC2, whereas it regulates glycolysis in Lin^+^ cells through both mTORC1 and mTORC2.

**Fig 5:**
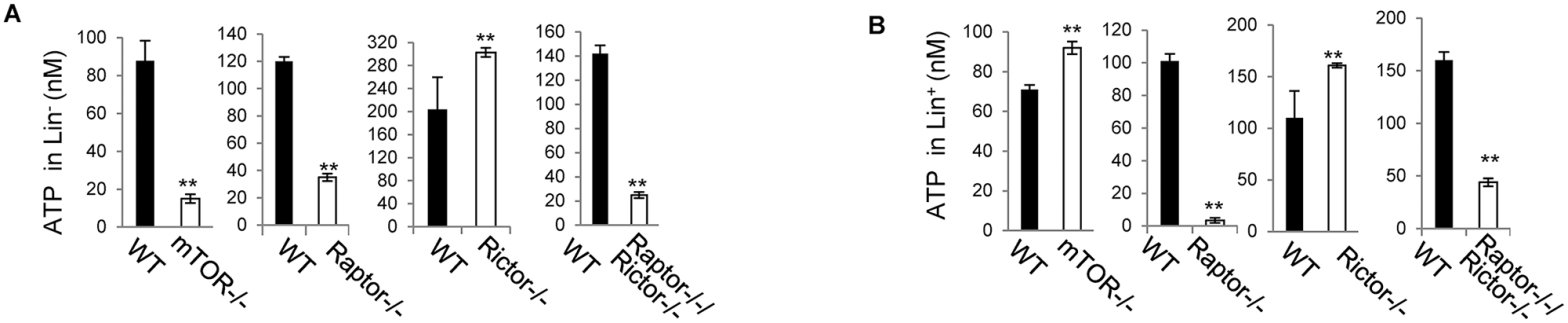
mTOR promotes energy production in Lin^-^ cells depending on mTORC1 but inhibits it in Lin^+^ cells independent of mTORC1 and mTORC2. Bone marrow cells were harvested from the indicated mice. Lin^-^ (A) and Lin^+^ (B) cells were fractionated from the bone marrow cells and assayed for ATP contents. Results are representative of three independent experiments. Error bars represent mean ± SD of 5–7 mice. **P < .01.

OXPHOS and glycolysis generate ATP. We found that ATP was decreased in *mTOR*^*-/-*^ Lin^-^ cells but increased in *mTOR*^*-/-*^ Lin^+^ cells (Fig 5A and 5B), suggesting that mTOR promotes energy production in Lin^-^ cells but inhibits it in Lin^+^ cells and thus mTOR plays a developmental stage-specific role in regulating energy production. Deletion of Raptor or simultaneous deletion of Raptor and Rictor, but not deletion of Rictor, recapitulated mTOR deletion in decreasing ATP in Lin^-^ cells (Fig 5A), suggesting that mTOR regulates energy production in Lin^-^ cells depending on mTORC1. In contrast to the increased ATP in *mTOR*^*-/-*^ Lin^+^ cells, ATP was decreased in *Raptor*^*-/-*^*Rictor*^*-/-*^ Lin^+^ cells (Fig 5B), suggesting that mTOR regulates energy production in Lin^+^ cells independent of mTORC1 and mTORC2. Interestingly, in both Lin^-^ and Lin^+^ cells, deletion of Raptor decreased ATP, whereas deletion of Rictor increased ATP (Fig 5A and 5B). These data suggest that mTORC1 and mTORC2 play an opposing role in regulating ATP production and that the effect of Raptor deficiency on ATP production prevails over that of Rictor deficiency, leading to a net decrease in ATP in *Raptor*^*-/-*^*Rictor*^*-/-*^ Lin^-^ and Lin^+^ cells. In addition, the decreased ATP in *mTOR*^*-/-*^, *Raptor*^*-/-*^ and *Raptor*^*-/-*^*Rictor*^*-/—*^Lin^-^ cells may reflect a combined effect of the decreased OXPHOS and glycolysis. The increased ATP in *mTOR*^*-/-*^ Lin^+^ cells may reflect a prevailing effect of the increased OXPHOS over the decreased glycolysis, whereas the decreased ATP in *Raptor*^*-/-*^ and *Raptor*^*-/-*^*Rictor*^*-/-*^ Lin^+^ cells may reflect a prevailing effect of the decreased glycolysis over the increased OXPHOS. On the other hand, the increased ATP in *Rictor*^*-/-*^ Lin^-^ and Lin^+^ cells can’t be explained by the decreased OXPHOS in *Rictor*^*-/-*^ Lin^-^ and the decreased glycolysis in *Rictor*^*-/-*^ Lin^+^ cells. It may reflect a net effect of OXPHOS, glycolysis and/or other metabolic programs such as lipid and amino acid metabolism.

### A role of mTOR, mTORC1 and mTORC2 in mitochondrial mass, mitochondrial membrane potential and mitochondrial DNA synthesis

**Fig 6:**
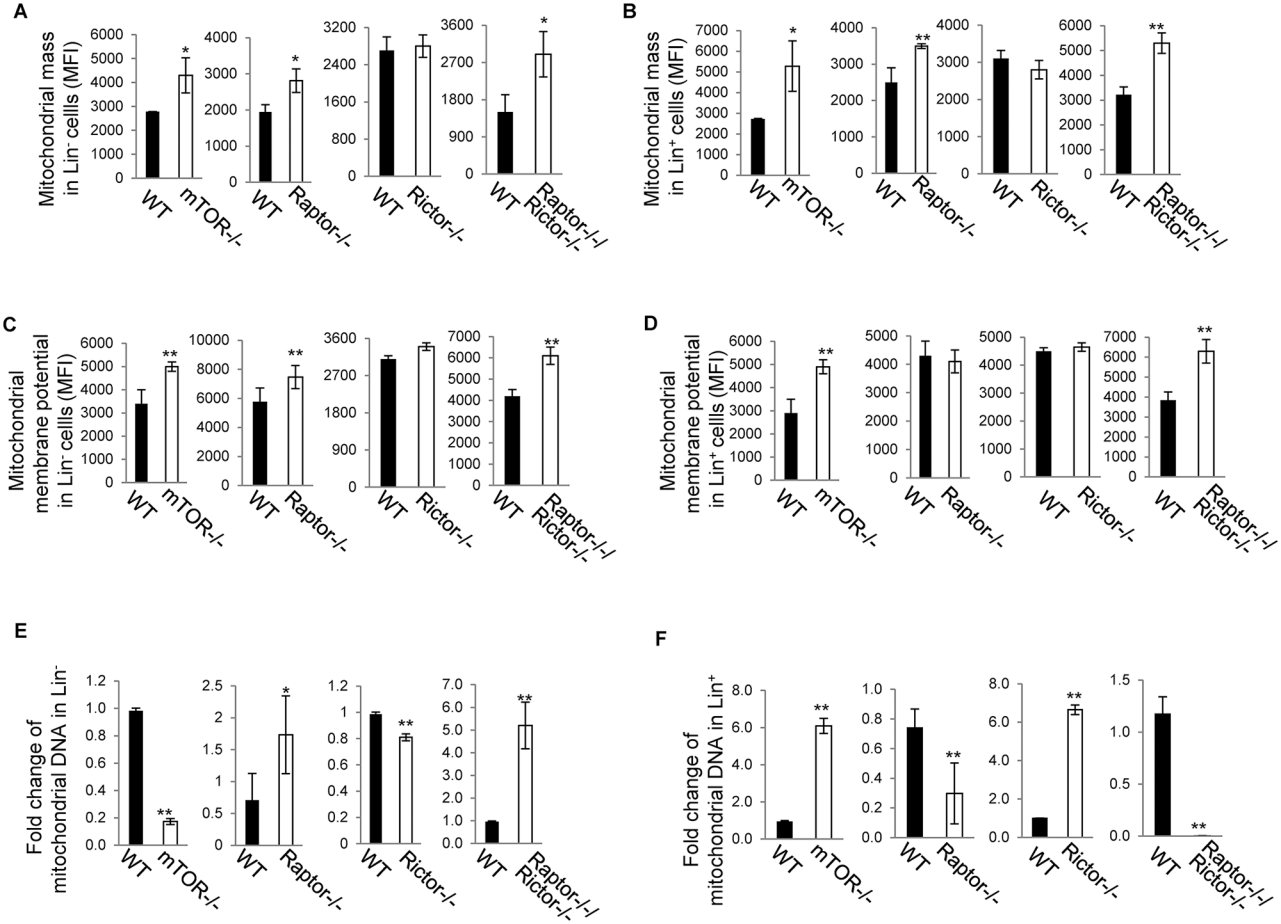
The effects of deletion of mTOR, Raptor and/or Rictor on mitochondrial numbers, mitochondrial membrane potential, and mitochondrial DNA synthesis. (A-D) Bone marrow cells were harvested from the indicated mice and immunolabeled with antibodies to lineage markers and with Mitotracker Green (A, B) or Dilc-5 (C, D). Mitochondrial numbers (A, B) and mitochondrial membrane potential (C, D) in Lin^-^ (A, C) and Lin^+^ (B, D) cells were then analyzed by flow cytometry. Mean fluorescence intensity (MFI) is shown. (E, F) Bone marrow cells were harvested from the indicated mice. Lin^-^ (E) and Lin^+^ (F) cells were fractionated from the bone marrow cells and assayed for Mitochondrial DNA contents. Mitochondrial DNA was represented by mitochondrial Cox 2 normalized to nuclear β-globin. The data are presented as mitochondrial DNA relative to one WT mouse. Results are representative of three independent experiments. Error bars represent mean ± SD of 5–7 mice. *P < .05, **P < .01.

Mitochondrial mass, mitochondrial membrane potential and mitochondrial DNA synthesis are important for mitochondrial metabolism. We found that depletion of mTOR or Raptor or simultaneous deletion of Raptor and Rictor increased mitochondrial mass in both Lin^-^ and Lin^+^ cells, whereas deletion of Rictor had no effect (Fig 6A and 6B), suggesting that mTOR maintains mitochondrial homeostasis in Lin^-^ and Lin^+^ cells dependent on mTORC1. Depletion of mTOR or Raptor or simultaneous deletion of Raptor and Rictor increased mitochondrial membrane potential in Lin^-^ cells, whereas deletion of Rictor had no effect (Fig 6C), suggesting that mTOR inhibits mitochondrial membrane potential in Lin^-^ cells dependent on mTORC1. Similar to that in Lin^-^ cells, depletion of mTOR increased mitochondrial membrane potential in Lin^+^ cells. However, while simultaneous deletion of Raptor and Rictor recapitulated mTOR deletion, deletion of Raptor or Rictor had no effect on mitochondrial membrane potential in Lin^+^ cells (Fig 6D). These results suggest that mTORC1 and mTORC2 play a redundant but important role in controlling mitochondrial membrane potential of Lin^+^ cells and that mTOR inhibits mitochondrial membrane potential of Lin^+^ cells depending on both mTORC1 and mTORC2. Deletion of mTOR decreased mitochondrial DNA in Lin^-^ cells but increased it in Lin^+^ cells (Fig 6E and 6F), suggesting a developmental stage-specific role of mTOR in regulating mitochondrial DNA synthesis. In contrast to mTOR deletion, simultaneous deletion of Raptor and Rictor increased mitochondrial DNA in Lin^-^ cells and decreased it in Lin^+^ cells (Fig 6E and 6F), suggesting that mTOR regulates mitochondrial DNA synthesis independent of mTORC1 and mTORC2. Similar to simultaneous deletion of Raptor and Rictor, deletion of Raptor increased mitochondrial DNA in Lin^-^ cells and decreased it in Lin^+^ cells (Fig 6E and 6F). In contrast to simultaneous deletion of Raptor and Rictor and deletion of Raptor, deletion of Rictor decreased mitochondrial DNA in Lin^-^ cells and increased it in Lin^+^ cells (Fig 6E and 6F). These data suggest that Raptor and Rictor play an opposing role in regulating mitochondrial DNA synthesis in Lin^-^ and Lin^+^ cells and that the effect of Raptor deficiency on mitochondrial DNA synthesis prevails over that of Rictor deficiency, leading to a net increase in mitochondrial DNA in *Raptor*^*-/-*^*Rictor*^*-/-*^ Lin^-^ cells and a net decrease in mitochondrial DNA in *Raptor*^*-/-*^*Rictor*^*-/-*^ Lin^+^ cells.

### A role of mTOR kinase activity in mitochondrial fitness

**Fig 7:**
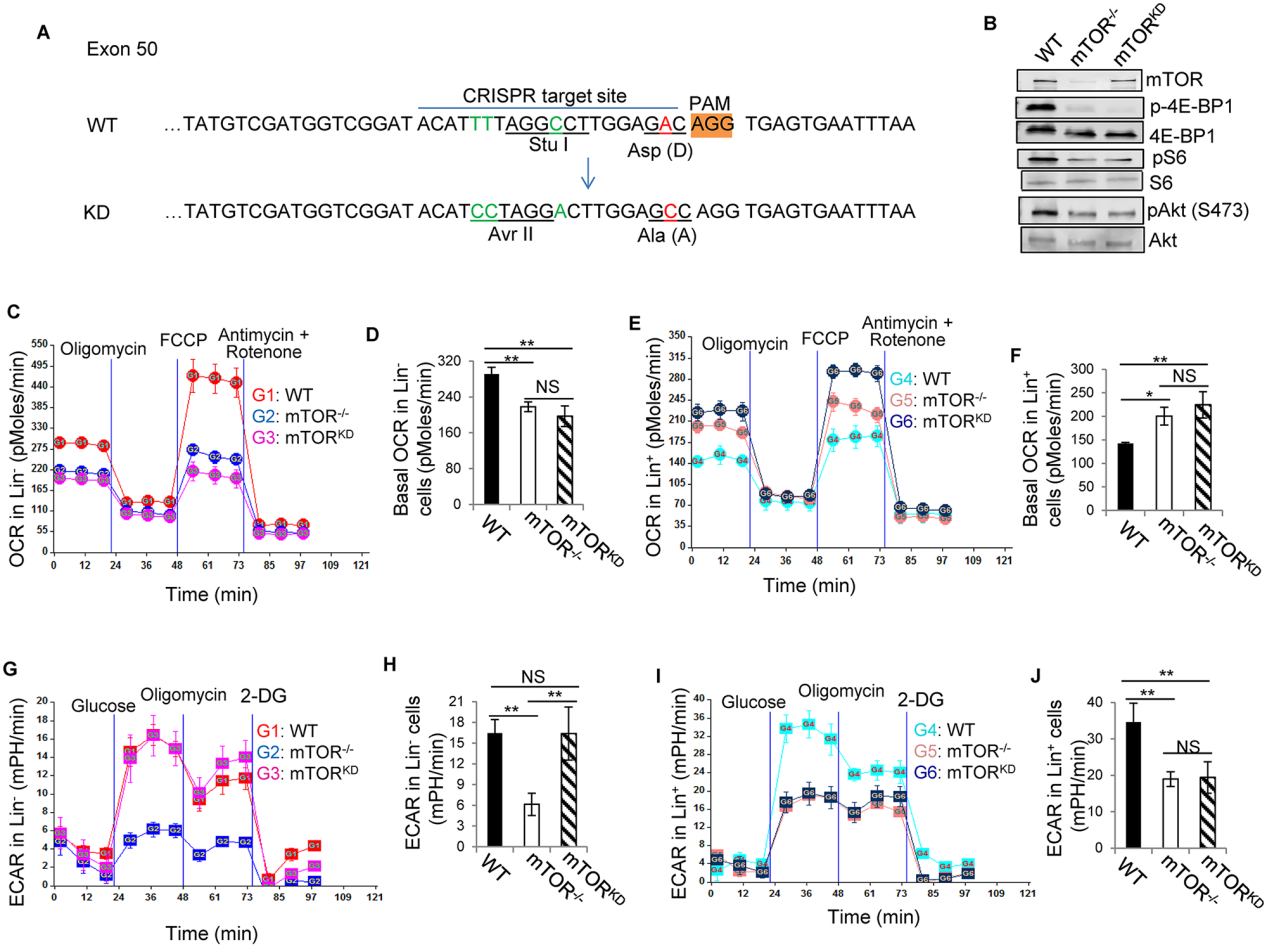
mTOR promotes glycolysis in Lin^-^ cells independent of its kinase activity. (A) mTOR kinase dead (KD) D2338A mutant knock-in strategy. mTOR KD mutant knock-in was achieved by CRISPR/Cas9 technology. Single guide RNA targeting site (CRISPR target site) is indicated. Amino acid 2338 of mTOR was changed from Asp (D) to Ala (A) (GAC to GCC). In addition, three silent mutations (highlighted in green) were introduced to abolish the restriction enzyme site of Stu I and create the restriction enzyme site of Avr II in the KD allele to facilitate genotyping of the mice (data not shown). The silent mutations also serve to prevent the CRISPR complex from re-cutting the KD allele. (B) Bone marrow cells were harvested from the indicated mice and detected for mTOR and phospho (p)-S6, 4E-BP and Akt by Western blot. Total 4E-BP, S6 and Akt were blotted as loading control. (C-J) Bone marrow cells were harvested from the indicated mice. Lin^-^ (C, D, G, H) and Lin^+^ (E, F, I, J) cells were fractionated from the bone marrow cells and assayed for oxygen consumption rate (OCR) (C-F) and extracellular acidification rate (ECAR) (G-J). OCR and ECAR profiles are shown in (C, E) and (G, I), respectively. Basal OCR in the absence of oligomycin, FCCP, and antimycin and rotenone from one measurement is shown in (D) and (F). ECAR in the presence of glucose but absence of oligomycin and 2-DG is shown in (H) and (J). Data are representative of three independent experiments. Error bars represent mean ± SD of 5–7 mice. *P < .05, **P < .01 determined by One-way ANOVA followed by Bonferroni test. mTOR^KD^: mTOR^loxp/KD^;Mx-Cre^+^. NS: no significance.

To define the role of mTOR kinase activity in mitochondrial fitness, we generated mTOR KD D2338A mutant [12, 19, 24] knock-in mice by CRISPR-Cas9 technology (Fig 7A). Because *mTOR*^*KD/KD*^ mice were embryonically lethal and *mTOR*^*+/KD*^ mice didn’t show abnormalities [19], we generated *mTOR*^*loxp/KD*^ mice and crossed with *Mx1-Cre*^+^ mice. When the resultant *mTOR*^*loxp/KD*^*;Mx1-Cre*^+^ mice are treated with poly I:C, mTOR is ablated from the floxed allele. Thus, mTOR kinase activity is not only lost on the D2338A mutant knock-in allele but also on the floxed allele, whereas protein expression is preserved on the D2338A mutant knock-in allele. By immunoblotting, we confirmed the preservation of mTOR protein expression and the loss of mTOR kinase activity in phosphorylating S6, 4E-BP and Akt in *mTOR*^*loxp/KD*^*;Mx1-Cre*^+^ bone marrow cells, upon poly I:C induction (Fig 7B). The loss of mTOR kinase activity recapitulated mTOR deficiency in decreasing basal and mitochondrial uncoupler FCCP-induced maximal OXPHOS in Lin^-^ cells and in increasing basal and maximal OXPHOS in Lin^+^ cells (Fig 7C–7F), suggesting that mTOR kinase activity is required for mTOR to regulate OXPHOS in Lin^-^ and Lin^+^ cells. The loss of mTOR kinase activity also recapitulated mTOR deficiency in decreasing glycolysis in Lin^+^ cells. However, it had no effect on glycolysis in Lin^-^ cells, in contrast to mTOR deficiency that caused decreased glycolysis in Lin^-^ cells (Fig 7G–7J). These data suggest that mTOR kinase activity is required for mTOR to regulate glycolysis in Lin^+^ cells but not in Lin^-^ cells.

## Discussion

In this study, by employing hematopoietic cells at the Lin^-^ and Lin^+^ stages of hematopoiesis as a model system, we demonstrate that mTOR has a development stage-specific role in the regulation of mitochondrial fitness, and mTOR regulates mitochondrial fitness in complex- and kinase activity-dependent and -independent manner. Specifically, direct comparison of the effects of mTOR deletion on mitochondrial fitness of Lin^-^ cells with that of Lin^+^ cells reveals that mTOR plays a development stage-specific role in regulating OXPHOS, ATP production and mitochondrial DNA synthesis (Table 1). By comparing the effects of mTOR deletion with that of simultaneous deletion of Raptor and Rictor on mitochondrial fitness, we show that mTOR regulates ATP production in Lin^+^ cells and mitochondrial DNA synthesis in both Lin^-^ and Lin^+^ cells independent of mTORC1 and mTORC2 (Table 2). It remains elusive how mTOR regulates ATP production and mitochondrial DNA synthesis in Lin^-^ and/or Lin^+^ cells. Nonetheless, given that mTOR can bind to IκB kinase (IKK) [25], IKK, in complex with mTOR, may underlie mTOR-regulated ATP production and mitochondrial DNA synthesis in Lin^-^ and/or Lin^+^ cells. By comparing the effects of mTOR deletion with that of Raptor or Rictor deletion on mitochondrial fitness of Lin^-^ and Lin^+^ cells, we demonstrate that mTOR regulates glycolysis, mitochondrial mass and mitochondrial membrane potential in Lin^-^ cells and mitochondrial mass and OXPHOS in Lin^+^ cells depending on mTORC1. In contrast, mTOR regulates OXPHOS in Lin^-^ cells and glycolysis in Lin^+^ cells depending on both mTORC1 and mTORC2 (Table 3). By comparing the effects of mTOR deletion with that of mTOR KD mutant knock-in on mitochondrial fitness, we unveil that mTOR regulates OXPHOS in both Lin^-^ and Lin^+^ cells and glycolysis in Lin^+^ cells depending on its kinase activity, whereas mTOR regulates glycolysis in Lin^-^ cells independent of its kinase activity.

**Table 1:** Changes in mitochondrial parameters in Lin^-^ and Lin^+^ cells upon deletion of mTOR.

Phenotype		Glycolysis	Mitochondrial mass	Mitochondrial membrane potential	OXPHOS	ATP	Mitochondrial DNA
Cell type							
**mTOR**^**-/-**^	**Lin**^**-**^	decrease	increase	increase	decrease	decrease	decrease
	**Lin**^**+**^	decrease	increase	increase	increase	increase	increase

**Table 2:** Changes in mitochondrial parameters in Lin^-^ and Lin^+^ cells upon deletion of mTOR or simultaneous deletion of Raptor and Rictor.

Phenotype		Glycolysis	Mitochondrial mass	Mitochondrial membrane potential	OXPHOS	ATP	Mitochondrial DNA
Cell type							
**Lin**^**-**^	**mTOR**^**-/-**^	decrease	increase	increase	decrease	decrease	decrease
	**Raptor**^**-/-**^**Rictor**^**-/-**^	decrease	increase	increase	decrease	decrease	increase
**Lin**^**+**^	**mTOR**^**-/-**^	decrease	increase	increase	increase	increase	increase
	**Raptor**^**-/-**^**Rictor**^**-/-**^	decrease	increase	increase	increase	decrease	decrease

**Table 3:** Changes in mitochondrial parameters in Lin^-^ and Lin^+^ cells upon deletion of mTOR, Raptor or Rictor.

Phenotype		Glycolysis	Mitochondrial mass	Mitochondrial Membrane potential	OXPHOS	ATP
Cell type						
**Lin**^**-**^	**mTOR**^**-/-**^	decrease	increase	increase	decrease	decrease
	**Raptor**^**-/-**^	decrease	increase	increase	decrease	decrease
	**Rictor**^**-/-**^				decrease	increase
**Lin**^**+**^	**mTOR**^**-/-**^	decrease	increase	increase	increase	increase
	**Raptor**^**-/-**^	decrease	increase		increase	decrease
	**Rictor**^**-/-**^	decrease				increase

One interesting observation is that while mTOR deficiency increases mitochondrial membrane potential in Lin^+^ cells, neither Raptor nor Rictor deficiency has an effect (Table 3). Consistent with our findings, it has previously been shown that while mTOR deficiency reduces muscle dystrophin and mTOR knockdown dampens translational efficiency of terminal oligopyrimidine (TOP) mRNAs, Raptor or Rictor deficiency shows marginal effect [12, 26]. It is seemingly true that mTOR regulates mitochondrial membrane potential in Lin^+^ cells, dystrophin expression in muscle cells and TOP mRNA translation independent of mTORC1 and mTORC2. However, the fact that simultaneous deletion of Raptor and Rictor recapitulates mTOR deficiency in increasing mitochondrial membrane potential in Lin^+^ cells (Table 2) rather suggests that mTOR depends on mTOR complexes in the regulation of mitochondrial membrane potential in Lin^+^ cells. These data also suggest that mTORC1 and mTORC2 play an important but redundant role in regulating mitochondrial membrane potential in Lin^+^ cells. Thus, whether mTOR functions through its complexes can only be revealed by comparing mTOR perturbation with simultaneous intervention of mTORC1 (e.g., Raptor) and mTORC2 (e.g., Rictor), and whether mTORC1 and mTORC2 are redundant but important for a cell function can only be revealed by comparing the intervention of mTORC1 or mTORC2 alone with simultaneous intervention of mTORC1 and mTORC2.

mTOR is a serine/threonine kinase and the kinase activity is expected to be responsible for its function. However, by knock-in an mTOR KD mutant, we show that mTOR regulates glycolysis in Lin^-^ cells independent of its kinase activity. By overexpression of the same mTOR KD mutant in muscle cells, a recent study has shown that mTOR has a kinase activity-independent role in regulating muscle dystrophin expression [12]. However, the non-specific nature of the overexpression approach may confound the conclusion of kinase activity independence of mTOR in regulating muscle dystrophin expression. In this context, our study unequivocally demonstrates that mTOR can function independent of its kinase activity in regulating mitochondrial fitness.

In conclusion, by using primary hematopoietic cells, our study has revealed that mTOR plays a developmental stage-specific role in the regulation of mitochondrial fitness. Furthermore, mTOR has a complex- and kinase activity-independent role in regulating mitochondrial fitness. Whether these observations can be extended to other cellular events and other tissues remains to be determined. Furthermore, as mitochondrial activity has been associated with hematopoietic cell behaviors [22, 27, 28], it will be interesting to see whether mTOR-regulated mitochondrial fitness has a role in hematopoiesis.

## Abbreviations

COX2: cyclo-oxygenase 2
ECAR: extracellular acidification rate
KD: kinase dead
Lin^-^: lineage-negative
Lin^+^: lineage-positive
mTOR: mammalian target of rapamycin
mTORC1: mTOR complex 1
mTORC2: mTOR complex 2
OCR: oxygen consumption rate
OXPHOS: oxidative phosphorylation
Poly I:C: polyinosine-polycytidine
SD: standard deviation
